# Performance and onsite regeneration of natural zeolite for ammonium removal in a field-scale non-sewered sanitation system

**DOI:** 10.1016/j.scitotenv.2021.145938

**Published:** 2021-07-01

**Authors:** C.J. Castro, H.Y. Shyu, L. Xaba, R. Bair, D.H. Yeh

**Affiliations:** a University of South Florida, Civil & Environmental Engineering, 4202 E. Fowler Ave, Tampa, FL 33620, USA; b Pollution Research Group, University of KwaZulu-Natal, Durban, South Africa

**Keywords:** Non-sewered sanitation, Zeolite, Ammonium, Wastewater, Resource recovery, Sorption

## Abstract

Natural zeolite clinoptilolite was used as the primary ammonium removal method from the permeate of an anaerobic membrane bioreactor (AnMBR) treating high-strength blackwater generated from a community toilet facility. This zeolite-based nutrient capture system (NCS) was a sub-component of a non-sewered sanitation system (NSSS) called the NEWgenerator and was field tested for 1.5 years at an informal settlement in South Africa. The NCS was operated for three consecutive loading cycles, each lasting 291, 110, and 52 days, respectively. Both blackwater (from toilets) and blackwater with yellow water (from toilets and urinals) were treated during the field trial. Over the three cycles, the NCS was able to remove 80 ± 28%, 64 ± 23%, and 94 ± 11%, respectively, of the influent ammonium. The addition of yellow water caused the rapid exhaustion of zeolite and the observed decrease of ammonium removal during Cycle 2. After Cycles 1 and 2, onsite regeneration was performed to recover the sorption capacity of the spent zeolite. The regenerant was comprised of NaCl under alkaline conditions and was operated as a recycle-batch to reduce the generation of regenerant waste. Modifications to the second regeneration process, including an increase in regenerant contact time from 15 to 30 h, improved the zeolite regeneration efficiency from 76 ± 0.7% to 96 ± 1.0%. The mass of recoverable ammonium in the regenerant was 2.63 kg NH_4_-N and 3.15 kg NH_4_-N after Regeneration 1 and 2, respectively. However, the mass of ammonium in the regenerant accounted for only 52.8% and 54.4% of the estimated NH_4_-N originally sorbed onto the zeolite beds after Cycles 1 and 2, respectively. The use of zeolite clinoptilolite is a feasible method for ammonium removal by NSSS that observe variable nitrogen loading rates, but further research is still needed to recover the nitrogen from the regenerant waste.

## Highlights

• 1.5 yr field trial of a zeolite-based nutrient capture system for ammonium removal.
• Three ammonium loading cycles of 291, 110, and 52 days were evaluated.
• Ammonium removal achieved for each cycle was 80 ± 28%, 64 ± 23%, and 94 ± 11%.
• Two onsite regenerations were conducted between cycles.
• Regeneration efficiencies for each regeneration were 76 ± 0.7% and 96 ± 1.0%.

## Graphical abstract

**Figure:**
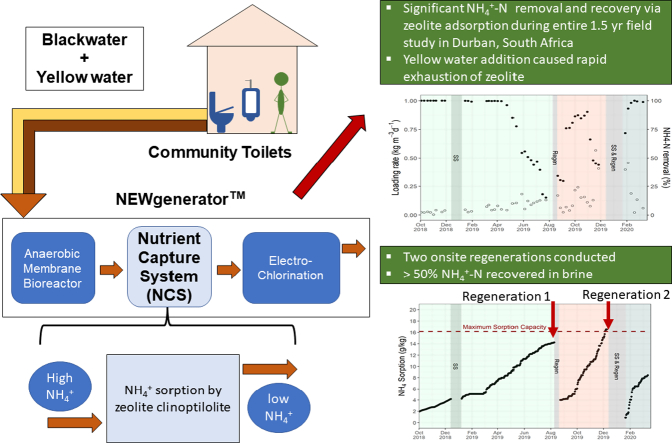

## 1 Introduction

Millions of people live in highly dense informal settlements that have limited or no access to connected sewers or sewage treatment (Baum et al., 2013). The lack of access to safely managed sanitation infrastructure propagates the practice of open defecation. Expanding global access to improved sanitation infrastructure is therefore interdependent with improving public health, hygiene, and water availability for over 4.2 billion people worldwide (UNICEF and WHO, 2019). Non-sewered sanitation systems (NSSS) are a form of decentralized wastewater treatment that collect, convey, and fully treat wastewater at the point of generation, thereby avoiding the costs and disruption of centralized sewer systems. NSSS are emerging solutions for improved wastewater management, particularly in developing countries experiencing rapid urbanization, with limited water resources, and inadequate sanitation infrastructure (de Feo et al., 2014).

While several NSSSs have been developed and tested in Africa and Asia, there has been a lack of uniform evaluation metrics, particularly relating to the technical maturity of a system and the reliability to manage by-products from exposure to people and the environment (Starkl et al., 2015). To standardize the evaluation of NSSS, The International Organization for Standardization (ISO) issued the ISO 30500 standards for designing and testing prefabricated NSSS in 2018 and defined the criteria for system performance and safety (ISO, 2018). The major effluent water quality parameters outlined by ISO 30500 consists of chemical oxygen demand (COD), total suspended solids (TSS), pH, pathogens, total nitrogen (TN), and total phosphorus (TP). While COD and TSS effluent standards have been met by several reported field-tested NSSS, meeting the ISO 30500 nutrient requirements consistently and for prolonged use has been challenging (Castro et al., 2014; Sahondo et al., 2020; Varigala et al., 2020; Shyu et al., 2021). The TN of blackwater is comprised of organic nitrogen, nitrate (NO_3_-N), nitrite (NO_2_-N), and ammonium (NH_4_-N), but NH_4_-N typically contributes to a significant portion of the TN (Gallagher and Sharvelle, 2009; Knerr et al., 2011). Without the benefits of dilution by graywater and other streams, the nutrient concentrations in blackwater (BW), defined as wastewater directly from toilets, can be nearly ten times higher than values observed at municipal wastewater treatment plants. The average values in BW are 35 mg/L for TP and 300 mg/L for TN, but concentrations can spike to much higher values during daily toilet use (Brandes, 1978; Cid et al., 2018; Knerr et al., 2011). NSSS should be designed to be robust enough to handle these extreme fluctuations in nutrient loading events caused by intermittent usage and dormancy periods, while maintaining simplicity in construction and operation.

For NH_4_-N removal, aerobic and anoxic biological nutrient removal (BNR) strategies have commonly been employed in centralized treatment plants. In these large-scale settings, BNR works very well because parameters such as dissolved oxygen (DO), pH, and oxidation/reduction potential (ORP) can be precisely monitored and controlled by operators, and where the costs of operation are generally non-prohibiting (Moore, 2009; Zhang et al., 2020). Newer methods, such as partial nitritation/anammox, have also been implemented at full-scale treatment plants to treat high strength nitrogen wastewater. However, performance is highly influenced by the varying influent solids concentration and maintaining precise DO levels in the system is critical (Lackner et al., 2014; Vlaeminck et al., 2009). For small-scale NSSS, aerobic and anoxic BNR processes have limited application due to their high energy demand for operation, the requirement of high precision control, the availability of reliable small-scale sensors for system monitoring, and the intermittent nature of NSSS (WSDH, 2005; Zhang et al., 2020). Furthermore, these BNR processes typically do not recover nitrogen for downstream reuse.

The challenges facing decentralized treatment systems have shifted the technological approaches to high-rate physical, chemical, and electrochemical treatment processes that can be operated more effectively in the field with limited process control (Bunce et al., 2018). Non-biological treatment methods such as air-stripping, breakpoint chlorination, ion exchange, and membrane separation are considered emerging technologies for NSSS applications (Trotochaud et al., 2020). For NH_4_-N separation, flat sheet nanofiltration/reverse osmosis (NF/RO) membranes have been tested but the NH_4_-N rejection is highly variable (61–93%) (Van Voorthuizen et al., 2005), limiting the technology for BW due to the high NH_4_-N feed concentrations. Cid et al. (2018) removed nitrogen by electrochemically producing free-chlorine and oxidizing ammonia to form chloramines, observing a decrease from 43 mg/L of Total Kjeldahl nitrogen (TKN) down to 5 mg TKN/L within 3 h. While successful at removing nitrogen from wastewater, the process itself is rather energy-intensive and does not recover nitrogen for reuse.

Zeolite, a microporous aluminosilicate mineral, has been extensively studied as an ion exchange material due to its high cation exchange capacity, with a particular affinity for NH_4_^+^, and efficient regenerative properties (Alshameri et al., 2014; Wijesinghe et al., 2016). The zeolite framework is comprised of layered channels of interlinking aluminosilicate tetrahedrons, creating open cages where ions and molecules can be captured (Kithome et al., 1998). Over 40 types of natural zeolites exist today, with clinoptilolite being the most abundant worldwide (Stefanakis et al., 2009; Flanagan and Crangle, 2020). For applications in NSSS, the use of zeolite clinoptilolite can be advantageous because it can handle high concentrations of NH_4_^+^, can adsorb between anywhere from 3 to 30 mg of NH_4_-N per gram of zeolite, and can be regenerated for subsequent reuse, with one study showing up to 20 regeneration cycles (Neag et al., 2019; Wasielewski et al., 2018; Zhang et al., 2017). The cost of zeolite varies by type, processing, and location but in general it ranges between $85–160 per ton, making zeolite an economically feasible technology for ammonia recovery (Flanagan and Crangle, 2020).

One of the major challenges of using zeolite as an NH_4_-N removal strategy is the chemical regeneration process, which typically involves the use of large quantities of NaCl and water to remove sorbed cations from the zeolite surface (Stone and Holliday, 1944). Similarly challenging is the lack of uniformity in the published data about how often zeolite can be regenerated and reused, which is widely dependent on the type of zeolite used and the composition of the ions present in wastewater (Guo et al., 2007; Katsou et al., 2011; Zhang et al., 2017). Many of the existing studies on regeneration have been conducted at the bench-scale in laboratory settings (Guida et al., 2020), which limits their direct translation to larger systems operating under ambient environmental conditions. To the best of our knowledge, there are no reported studies on onsite NH_4_-N removal performance using zeolite or field regeneration when treating BW using NSSS. Field studies of regeneration processes are critical in evaluating and improving system operating protocols. With support from the Bill and Melinda Gates Foundation through the “Reinvent the Toilet Challenge,” a wastewater treatment technology called the NEWgenerator™ (NG) was deployed and operated for 1.5 years in an informal settlement in the eThekwini Municipality of South Africa (Shyu et al., 2021). The performance was evaluated against the ISO 30500 water quality standards for NSSS. The NG was able to meet COD, TSS, and pH effluent requirements for category B water reuse, as outlined by ISO 30500, as well as achieving over 6 log removals of *E. coli*. The NG incorporated a nutrient capture system (NCS) with natural zeolite as the main NH_4_^+^-N treatment strategy and, on average, was able to meet the 70% total nitrogen reduction requirement. The purpose of this paper is to report on the NH_4_-N removal performance of the full-scale zeolite-based NCS used within the field-tested NG. The study also highlights the onsite field regeneration process and the performance of the zeolite beds during subsequent regeneration cycles.

## 2 Materials and methods

### 2.1 NCS configuration

The NEWgenerator used in this field study was a modified version of a previously field-tested treatment system in India, NG-100 v.1, which used an anaerobic membrane bioreactor (AnMBR) and an electrochlorinator (EC) as the primary treatment processes (Bair, 2016). The India NG-100 v.1 consisted of a vertical hydroponics system and reused available nutrients from the effluent of the AnMBR to support the growth of plants. While the permeate is suitable as a fertilizer for plants and crops (fertigation), not all communities desire this end use product. Furthermore, to meet environmental discharge or water reuse requirements for purposes such as flush water, ISO 30500 requires that 70% of TN be removed from the effluent. Therefore, the modified version NG-100 v.2, from here on referred to as NG, had three main treatment steps as previously described by Shyu et al. (2021) to yield effluent water safe for discharge or reuse. In brief, the system consisted of a three-chamber AnMBR, a NCS, and EC for polishing of the finished water.

**Fig. 1:**
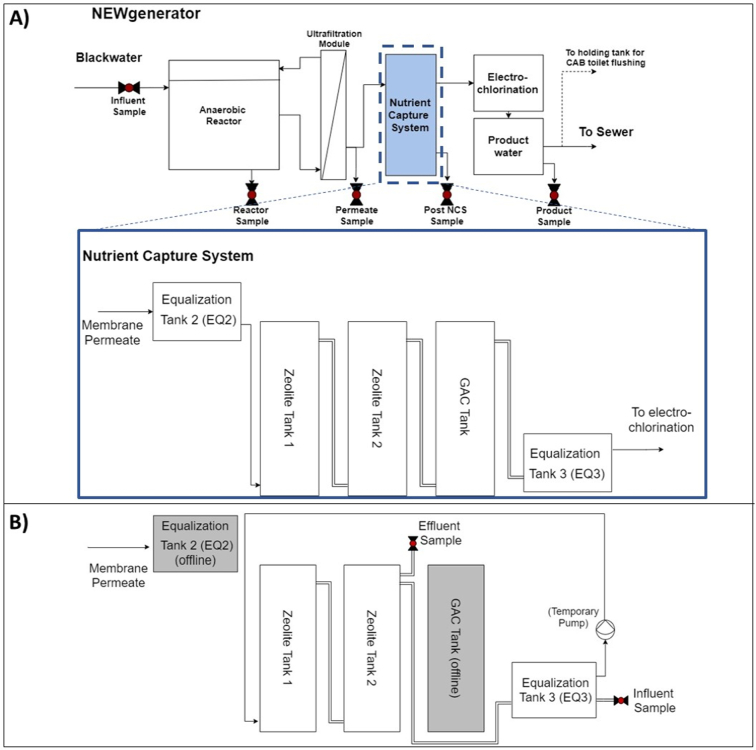
Flow diagram of the NCS during A) normal field trial operations and B) the regeneration process.

Permeate from the AnMBR was pumped into an equalization tank (EQ2) and then gravity-fed into the NCS. The NCS consisted of three hydraulically connected rectangular 360 L tanks (Fig. 1A). The first two tanks had an effective liquid volume of 250 L and each contained 175 kg of locally sourced natural zeolite clinoptilolite with an average diameter of 0.85–2 mm. The third tank contained 80 kg of granular activated carbon (GAC) to remove residual color and soluble COD. The hydraulic retention time (HRT) of the NCS system was designed to be 12 h but this varied based on the overall system's daily flow rates, which ranged anywhere between 37 and 715 L/d on any given day. The effluent of the NCS entered another equalization tank (EQ3) before being pumped to the EC system for disinfection and final polishing. Water quality samples were taken at the influent, in the reactor of the AnMBR, from the permeate exiting the membrane, in EQ3, and after chlorination.

### 2.2 System operation

**Fig. 2:**
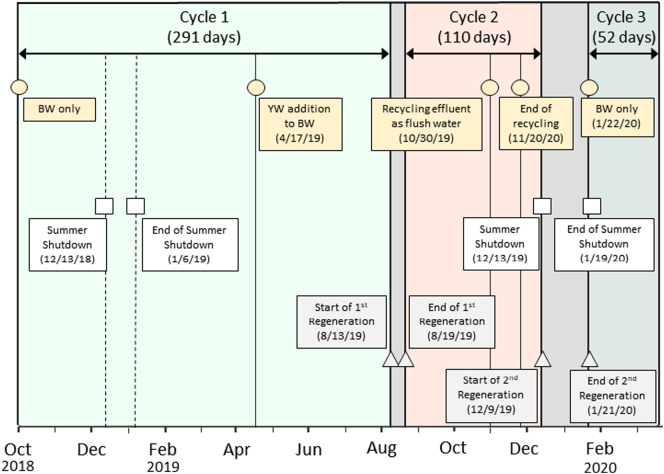
Timeline of events during the field trial. Circles represent time points when the influent media changed; squares represent scheduled shutdown periods when UKZN staff were on summer holiday; and triangles represent the start and end of regeneration events. The operational time is divided into three cycles: Cycle 1 (green shaded region), Cycle 2 (pink shaded region), and Cycle 3 (blue shaded region). (For interpretation of the references to color in this figure legend, the reader is referred to the web version of this article.)

The NG was field-tested for over 1.5 years at an informal settlement in eThekwini Municipality, South Africa. The system's operation and performance were described by Shyu et al. (2021). In that study, the field trial's operational time was divided into three stages, each defined by the strength and type of wastewater entering the system. NG first treated BW, which consisted only of flushed toilet water from a community ablution block (CAB) for male users. The next stage treated BW along with yellow water (BW + YW), where the yellow water was undiluted urine from the CAB's waterless urinals. In the final stage, NG again treated BW only. Since the purpose of this study was to evaluate the NH_4_-N removal and recovery by zeolite in the NCS regardless of wastewater type, the total field trial operation was divided into three loading cycles (Cycle 1: Oct. 1, 2018–Aug. 12, 2019, 291 days; Cycle 2: Aug. 17, 2019–Dec. 8, 2019, 110 days; Cycle 3: Jan. 22, 2020–Mar. 13, 2020, 52 days), separated by two zeolite regeneration events. The duration of each cycle included weekends and short maintenance events when the system was off-line. Fig. 2 summarizes the timeline of major events and shutdown periods during this study. Samples from the five sampling locations were taken weekly (unless otherwise noted), stored at 4 °C and analyzed for TN, total ammonia nitrogen (TAN), NO_3_-N, conductivity, and pH. Since the pH was well below the pKa of ammonia (9.3) TAN is denoted as ammonium‑nitrogen (NH_4_-N) within the system unless otherwise stated.

### 2.3 NCS regeneration process

#### 2.3.1 Regeneration 1

The concentration of NH_4_-N was monitored at all sampling points on a weekly basis. Once NH_4_-N breakthrough was observed, it was intentionally allowed to continue to near saturation for the purpose of determining the NH_4_-N field uptake capacity by the NCS before regeneration of the zeolite beds was performed. The first regeneration event occurred on Aug 13–16, 2019, marking the end of Cycle 1. The NG was stopped completely and the two zeolite tanks of the NCS were hydraulically disconnected from EQ2 and the GAC tank. The zeolite tanks were emptied of liquid content, rinsed once with tap water, and emptied again prior to beginning the regeneration process. The process flow diagram for regeneration is shown in Fig. 1B. A high concentration of Na^+^ ions was used as the primary regenerate cation due to the higher selectivity of Na^+^ over NH_4_^+^ by zeolite. The regeneration solution (60 g NaCl/L) consisted of 36 kg NaCl dissolved in approximately 600 L of tap water. The NaCl concentration was chosen to ensure complete desorption of NH_4_-N for an anticipated maximum capacity of 20 mg NH_4_-N per g of zeolite. The regeneration solution was recirculated between the zeolite tanks and EQ3 for 5 h each day over three days and left soaking overnight. Recirculation of the regenerant was done to promote adequate mixing and to minimize the overall liquid volume of regenerant utilized. Furthermore, since the desorbed NH_4_^+^ ions would be recirculated back to the zeolite beds, the pH of EQ3 was continuously measured and adjusted to pH 10 in real-time while the liquid content of the tanks was recirculated in order to transform NH_4_^+^ to NH_3_. The total contact time with NaCl was 56 h (15 h of recirculation and 41 h of static soaking). Liquid samples of 50 mL were collected during working hours from the influent and effluent of EQ3. Samples were analyzed for TAN, Cl^−^, Na^+^, and pH. The liquid samples collected in the field were stored in a cooler and returned to the lab for processing by the end of each sampling day. Samples were analyzed within 3–5 days of collection by the Pollution Research Group (PRG) operating within the University of KwaZulu-Natal (UKZN) in Durban, South Africa. After the regeneration was completed, the beds were rinsed with 4 total bed volumes of tap water and deemed ready for Cycle 2 operation. To determine the regeneration efficiency, a 1 L sample of zeolite granules was collected from the top 5 cm of each bed after the final rinse for analysis.

#### 2.3.2 Regeneration 2

The second regeneration was coupled with the 6-week summer holiday break of the PRG research lab from December 9, 2019 to January 21, 2020, during which the field engineers were not on site to operate the NG. Coupling the regeneration with the break was done to reduce the down time of the system during other times of the year. During the second regeneration, the zeolite tanks were drained and rinsed as previously described in the first regeneration. Several modifications were made to the second regeneration protocol. The regenerant concentration was increased to 66.7 g NaCl/L (40 kg NaCl and dissolved in approximately 600 L of tap water). The pH of EQ3 was measured and adjusted to pH 10 while the liquid content of the tanks recirculated. The regeneration solution was recirculated between the zeolite tanks and EQ3 for 6 h on the first day and 24 h on the second day, with the regenerate solution left soaking in between recirculation. Therefore, the recirculation time of the regenerate solution was increased to 30 h with 18 h of soaking. Then, the pump was turned off and the tanks were left soaking in the regeneration solution for the remainder of the summer shutdown for an additional 38 days. Afterwards, the tanks were each consecutively rinsed with tap water for 10 bed volumes before Cycle 3 operation of the NG began. Liquid samples were taken from EQ3 after the initial 48 h of recirculation and soaking the regeneration solution, after 38 days of soaking, and after the final rinse of the zeolite tanks. Samples were analyzed for TAN, turbidity, and pH. Zeolite granules were again collected 5 cm from the top of each bed after the final rinse to determine the regeneration efficiency.

### 2.4 Data analysis & calculations

#### 2.4.1 Ammonium loading rate

TN, NH_4_-N, NO_3_-N, Cl^−^, and Na^+^ were all processed by the PRG lab using Merck kits following standard methods. Conductivity and pH were measured in the lab using HACH pH and conductivity probes while turbidity was measured using a HACH portable turbidity meter. The pH in the field was measured using a Hanna HI98190 portable probe. The processed water delivered by NG was measured on a daily basis using an analog flow meter located after the product tank. The NH_4_-N loading rate, *L_NH4_* (kg m^-3^ d^-1^), into the NCS was determined using the following equation:

(1) $$L_{NH_{4}}=\frac{C_{\mathrm{perm}}\times Q_{\mathrm{avg}}}{V_{z\left(l\right)}}$$

where the C_perm_ was the observed daily concentration in the permeate sample in kg/L, Q_avg_ was the average daily flow rate between two samples in L/d, and V_z(l)_ was the combined liquid volume of the zeolite tanks in m^3^ and estimated to be 0.50 m^3^.

#### 2.4.2 Ammonium sorption by zeolite

To determine the maximum NH_4_-N capacity of the zeolite used in this study, 3 g of zeolite granules were soaked in a 1 M NH_4_Cl solution for 336 h to fully saturate the zeolite. After the zeolite was saturated, 2 g of granules were rinsed with deionized water and agitated in 100 mL of 5% HCl solution for 24 h to desorb the NH_4_-N. The NH_4_-N concentration in solution was measured and the zeolite granules were rinsed and dried at 150 °C before weighing. All tests were done in triplicates. The NH_4_-N sorption capacity, q (g N/kg), during the batch test was calculated using the following equation:

(2) $$q=\frac{\left(C_{o}-C\right)\times V}{w_{z}}$$

where the C_o_ was the initial concentration of NH_4_^+^ as g NH_4_-N/L, C was the final concentration of NH_4_-N as g/L, V was the volume of the solution in L, and w_z_ was the weight of the zeolite in kg. The final maximum laboratory NH_4_-N sorption capacity of the zeolite, q_max_, was 16.17 ± 0.13 g/kg. To determine the daily NH_4_-N sorption during the field trial, C_o_ was the influent concentration entering the NCS as g N/L, C was the effluent concentration exiting the NCS as g N/L, the V was the volume of wastewater processed in L, and w_z_ was the weight of the zeolite in the tanks (350 kg). The total NH_4_-N sorbed between regenerations was estimated by the following equation:

(3) $$q_{\mathrm{TOT}}=q_{o}+\sum_{i=1}^{k}\frac{{\left(C_{\mathrm{perm}}-C_{\mathrm{NCS}}\right)}_{i}\times V_{i}}{w_{z}}$$

where q_TOT_ was the amount of NH_4_-N sorbed by a specific day in g N/kg, q_o_ was the residual mass of NH_4_-N sorbed onto the zeolite in g/kg, C_perm_ was the NH_4_-N concentration in the permeate in g N/L, C_NCS_ was the concentration of NH_4_-N in NCS effluent, V_i_ was the volume of water processed in L, and k was the number of days operated before regeneration. Since the NG began intermittent operations as part of the startup in July 2018 but the collection of water quality data began in October 2018, the residual NH_4_-N uptake by the NCS during the startup period, q_o_, needed to be estimated. It was assumed that the NG was operated for 60 working days prior to the start of data collection under normal operations. It was also assumed that the water production volumes remained linear to the production rate of Cycle 1 and that the concentration of NH_4_-N was on average 85 mg N/L, equivalent to the average concentration during the first three months of BW treatment operation. Lastly, since 100% uptake of NH_4_-N was observed during the first 5 months of water quality collection, it was assumed that all NH_4_-N entering the NCS would be sorbed by zeolite. The q_TOT_ after each loading cycle was calculated to be 14.2, 16.5, and 8.4 kg/g, respectively.

#### 2.4.3 Regeneration efficiency

To determine the regeneration efficiency during each regeneration event, 100 g of zeolite granules were collected from each of the zeolite beds after regeneration occurred and stored at 4 °C. The sorbate amount on the zeolite sample was determined as previously described. The efficiency was calculated using the following equation:

(4) $$\eta_{\mathrm{regen}}=\left(1-\frac{q_{z}}{q_{\mathrm{TOT}}}\right)*100$$

where η_regen_ was the regeneration efficiency and q_z_ was the residual NH_4_-N that remained sorbed onto the zeolite in g/kg. Efficiency values are presented as percentages.

## 3 Results

### 3.1 Nitrogen treatment performance

**Fig. 3:**
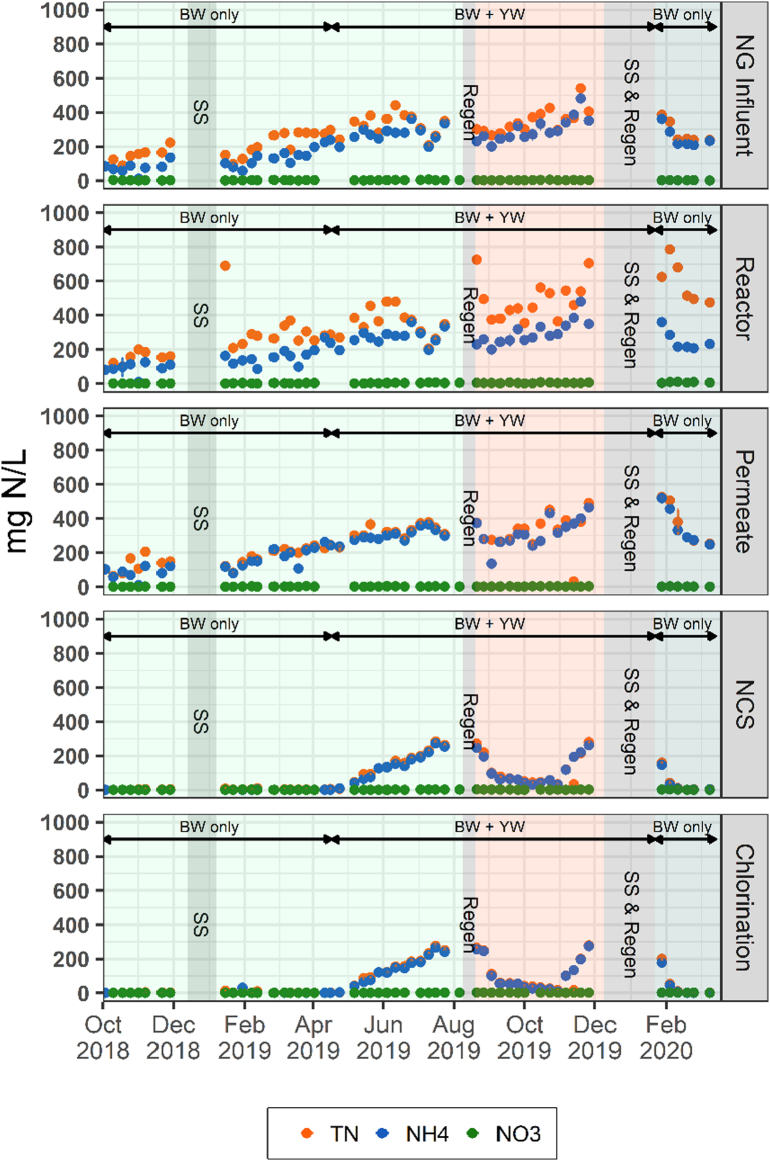
Concentration of nitrogenous species (total nitrogen, ammonium, and nitrate) measured across the entire field trial. The operational time is divided into three cycles: Cycle 1 (green shaded region), Cycle 2 (pink shaded region), and Cycle 3 (blue shaded region). Note: Summer shutdown (SS) and Regeneration events (Regen). TN data for the NG influent and after Chlorination are reproduced from Shyu et al. (2021). (For interpretation of the references to color in this figure legend, the reader is referred to the web version of this article.)

To characterize the major nitrogen species passing though the NG, the concentrations of TN, NH_4_-N, and NO_3_-N were measured across the entire field trial beginning from October 2018 until March 2020, except for the summer shutdown periods, during regeneration events, and during a brief break in February 2019 due to conference travel (Fig. 3). Overall, all concentrations of nitrogen species in the influent, reactor, and permeate began to increase after the first summer break in 2018 likely due to biodegradation of particulate solids in the influent equalization tank and in the reactor tank. This increase stabilized around March 2019, taking nearly a month to reach a stable baseline. While BW was used during the entire field trial, from mid-April to the end of November 2019, YW from the CAB urinals was added to the BW liquid stream. The addition of YW during this time period caused an observable increase in TN in the influent and in the reactor. This increase indicated a spike in organically-bound nitrogen from the addition of urine. A large increase in TN in the reactor also occurred during Cycle 3 when the system was restarted after a 6-week shutdown. This increase was likely due to an accumulation of organic nitrogen in the reactor contributed from organically-bound nitrogen being solubilized from the YW prior to shutdown. While nitrogen levels were high in the reactor, the membrane was able to effectively retain the organic nitrogen during the entire field trial, as observed by the convergence of TN and NH_4_-N concentrations to near equal values in the permeate samples. Inorganic nitrogen species were therefore the primary nitrogen species downstream of the membrane for all cycles.

The average nitrate concentrations entering NG during the field trial remained relatively low, below 2.5 mg N/L (Table S1). An increase in nitrate was observed in the reactor for all cycles, similar to what was observed with total nitrogen. These elevated concentrations all decreased to 1.4–1.9 mg N/L in the permeate, suggesting that denitrification activity may have reduced nitrate levels in the anaerobic reactor. Finally, very little to no change was observed in nitrate concentrations in the NCS and chlorinator effluents during all three cycles, bringing the final NG effluent values to an average of 0.60, 1.6, and 1.6 mg NO_3_-N/L, respectively. The nitrate concentration of TN was insignificant when compared to NH_4_-N, accounting for no more than 1% of the total measured nitrogen in the system. Therefore, it can be assumed that the NH_4_^+^ was equivalent to the total nitrogen after membrane filtration.

The average NH_4_-N concentrations remained stable, spatially, between the influent, reactor, and the permeate samples. As expected, the primary NH_4_-N removal mechanism was by sorption in the NCS. No further change was observed in the NH_4_-N concentration due to chlorination. The NCS alone was able to remove, on average, 80 ± 28%, 64 ± 23%, and 94 ± 11% of all influent NH_4_-N in Cycles 1, 2, and 3, respectively. However, temporally, the NH_4_-N levels across the system were highly influenced by the type of wastewater being treated. After YW addition, a steady increase in NH_4_-N was observed in the NCS effluent over several weeks. Intentionally, NH_4_-N breakthrough was allowed to continue for the purpose of determining the NH_4_-N field sorption capacity by the NCS and to test the regeneration procedure on a near saturated zeolite bed. After the first regeneration of the zeolite tanks, NH_4_-N began to decrease in the NCS samples, indicating a successful regeneration. A similar trend was observed before and after the second regeneration. During the regeneration events, the GAC bed was not included as part of the regeneration process, meaning that the liquid held within the GAC tank influenced the initial nitrogen values observed in EQ3 immediately after restarting NG.

The influent and reactor NH_4_-N concentrations remained steady even after the YW addition, until 30 October 2019, when the system began to recycle effluent water to the CAB for flushing toilets for a brief period of 16 working days. During this brief period, the NCS observed a rapid breakthrough in NH_4_-N, indicating that the zeolite tanks were becoming saturated. During this breakthrough, NH_4_-N concentrations reached a maximum of 270 mg N/L in the final NG effluent, similar to the average influent concentration of Cycle 2. The combination of recycling product water that contained high levels of NH_4_-N coupled with a partially exhausted NCS caused a rapid accumulation of NH_4_-N within the system and a spike in observed concentration. After the second shutdown and regeneration, YW from the CAB urinals was diverted to sewer prior to starting Cycle 3. During Cycle 3, NH_4_-N concentrations decreased and stabilized in the influent, reactor, and permeate samples. With a newly regenerated NCS, the NH_4_-N concentrations in the post NCS samples were near zero after a three-week stabilization period.

### 3.2 Zeolite exhaustion

#### 3.2.1 Ammonium loading rates

**Fig. 4:**
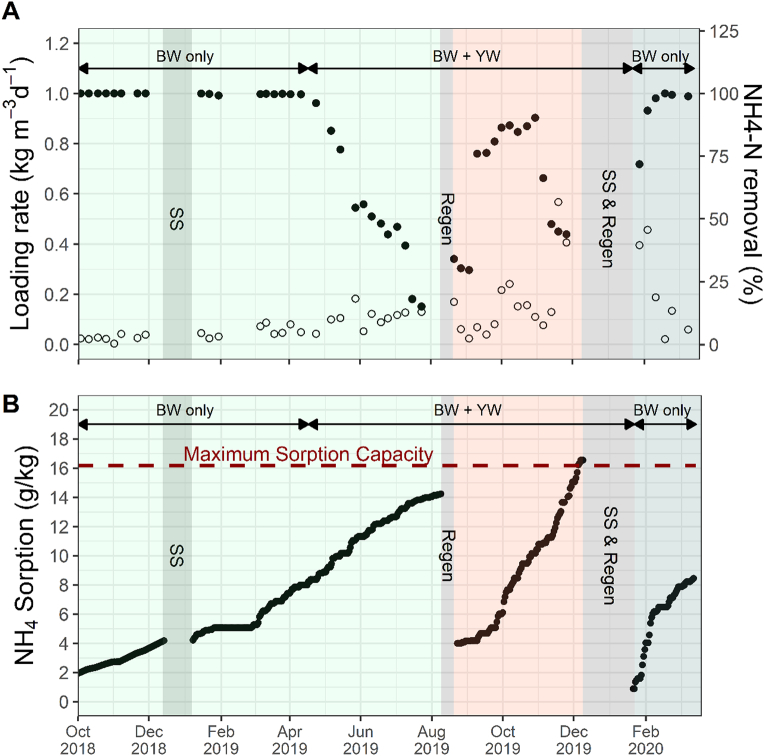
A) Ammonium loading rate into the zeolite beds (unfilled circle) and percent ammonium removal by the NCS (black circle) and B) ammonium sorption by zeolite beds of the NCS during the entire field trial. The laboratory estimated maximum ammonium sorption capacity of the zeolite used in this study was 16.2 g/kg.

The NH_4_-N loading rate into the NCS observed similar fluctuations and increases as the NH_4_-N concentrations (Fig. 4A). In Cycle 1, the NH_4_-N loading rate was consistently below 0.09 kg m^−3^ d^−1^ and increased to 0.11 ± 0.04 kg m^−3^ d^−1^ when YW was added to the wastewater influent. In Cycle 2, the loading rate was more variable and loads as high as 0.57 kg m^−3^ d^−1^ were observed prior to the second regeneration cycle. Finally, in Cycle 3, NH_4_-N loading rates decreased and stabilized to levels similar to those observed in Cycle 1.

Ammonium removal within the NCS was maintained at near 100% until loading rates began to increase after YW addition, in which the percent removal decreased down to 15% until a regeneration event occurred (Fig. 4A). The increased loading rates after YW addition contributed to the rapid saturation of the zeolite beds at the end of Cycle 1 and 2, indicated by the drop of NH_4_-N removal performance. After the first regeneration event, the zeolite tanks were able to effectively handle the variable nitrogen and volumetric loads, though the increase in nitrogen loading rates led to faster saturation of the zeolite in cycle 2. After the second regeneration, the removal percent in Cycle 3 was able to recover more rapidly than in the first regeneration.

#### 3.2.2 Ammonium sorption

During Cycle 1, the zeolite beds adsorbed 14.2 g/kg before regeneration of the zeolite beds was conducted. During Cycle 2, the zeolite beds reached an sorption capacity of 16.5 g/kg before regeneration of the beds (Fig. 4B). From laboratory tests, the maximum sorption capacity of NH_4_-N was 16.2 ± 0.13 g/kg. The rate of NH_4_-N sorption was dependent on the daily volume of water processed by the system as well as the increase in NH_4_^+^ loading rates during the second half of Cycle 1 and all of Cycle 2. The observed flow rates during each cycle were 116 (R^2^ = 0.99), 190 (R^2^ = 0.96), and 143 (R^2^ = 0.92) L/d, with Cycles 2 and 3 having the most fluctuations in flow rate as observed by the NH_4_-N sorption rate. The NH_4_-N sorption rate by zeolite was on average 0.06 g/kg-d in Cycle 1 and increased two-fold in Cycle 2 to 0.11 g/kg-d. In Cycle 2, overnight operation of NG became more common in order to increase the volume of product water generated. During Cycle 3, flow rates increased significantly during the first two weeks due to longer run times to replenish the nutrient capture system's liquid volume after restarting the system, observing sorption rates of 0.31 g/kg-d and then decreasing to 0.08 g/kg-d until the end of the field trial. When the NH_4_-N sorption capacity in the NCS reached 11.0 g/kg, or 1.1%, in either Cycle 1 or 2, the percent removal of NH_4_-N dropped below 70%.

### 3.3 Zeolite regeneration

**Fig. 5:**
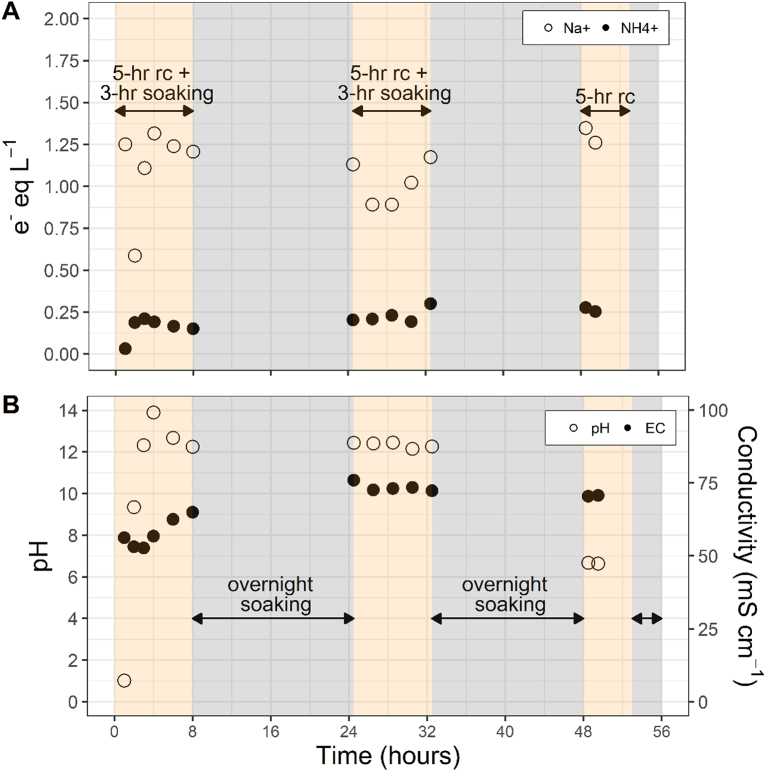
A) Electron equivalents of ammonium and sodium and B) conductivity and pH measured in the regenerate solution during Regeneration 1. Note: recirculation period (rc) and grey areas represent periods of static and/or overnight soaking only.

**Fig. 6:**
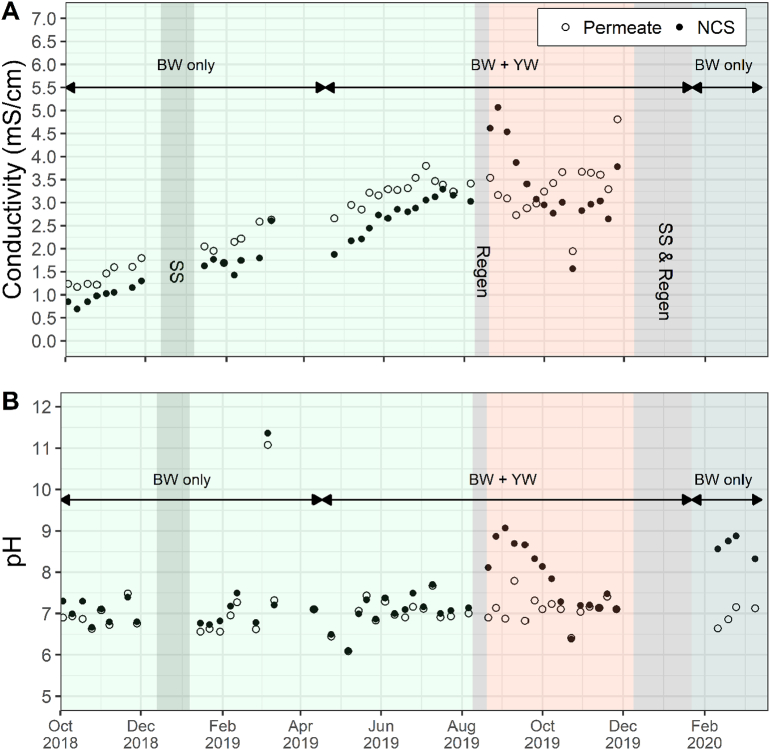
A) Conductivity and B) pH of the influent (permeate) and effluent (NCS) of the NCS during entire field trial. No conductivity data is available for Cycle 3.

During the first regeneration, NH_4_-N, Na^+^, Cl^−^, turbidity, conductivity, and pH were measured in the regenerate solution during the recirculation period. The regenerate solution contained an excess of Na^+^ ions to ensure that exchange ions were available for a maximum sorption up to 20 g/kg. The electron equivalents of NH_4_-N in the regenerate solution reached an equilibrium of 0.24 ± 0.04 e^−^ eq/L (3.41 ± 0.57 g NH_4_-N/L) while Na^+^ decreased from 1.3 to 0.89 e^−^ eq/L after 8 h of recirculation and 16 h of soaking (Fig. 5A). Similarly, the Cl^−^ concentration stayed relatively unchanged, remaining at 1.09 ± 0.12 after 24 h of operation (Fig. S1). The pH remained at 10.2 ± 0.3 during this same time frame (Fig. 5B). Turbidity was also measured and remained below 2.0 NTU after regeneration was completed (Fig. S1). The maximum amount of NH_4_-N anticipated in the regenerate, based on the estimated sorption during Cycle 1, was 0.59 e^−^ eq/L. The pH of the regenerate solution was maintained higher than the ammonia pKa (9.25), suggesting that much of the unaccounted NH_4_-N in solution may have stripped out as NH_3(g)_ during recirculation, although aeration was not actively performed. The partial loss of NH_4_-N was evident when the operator noted the smell of ammonia as well as the minor decrease in regenerate conductivity over the course of the process (Fig. 5B). Following the first regeneration event, residual NaCl and NaOH from the regenerate solution persisted within the NCS, also evident by the large spikes in conductivity and pH in the effluent (NCS sample) immediately after the regeneration event occurred (Fig. 6).

The results from Regeneration 1 were used to anticipate the performance of Regeneration 2. The second regeneration process followed a modified protocol when compared to the first regeneration: increased Na^+^ from 23.6 g/L to 26.2 g/L, the regenerate recirculation time increased from 15 h to 30 h, and the beds had an additional soaking period of 38 days. The event was coupled with the summer shutdown period and the zeolite spent nearly 6 weeks soaking in regenerate solution. The first regeneration had no extended soaking time immediately after the 56-hr contact time and resulted in 76 ± 0.7% zeolite regeneration between the two beds (Table 1). On the other hand, the additional contact time in the second regeneration improved the results to 96 ± 1.0%.

**Table 1:** Summary of regenerant contact time and residual ammonium adsorption on final zeolite samples after the regeneration process was complete.

	**Regeneration 1**		**Regeneration 2**	
	Bed 1	Bed 2	Bed 1	Bed 2
Recirculation Time (hrs)	15	15	30	30
Overnight Soaking (hrs)	41	41	18	18
Extended Soaking (days)	0	0	38	38
Residual NH_4_-N adsorbed (g/kg)	3.0 ± 0.06	4.9 ± 0.20	0.50 ± 0.20	0.80 ± 0.20
Regeneration Efficiency (%)	82 ± 0.40	70 ± 0.91	97 ± 1.1	95 ± 0.95

**Fig. 7:**
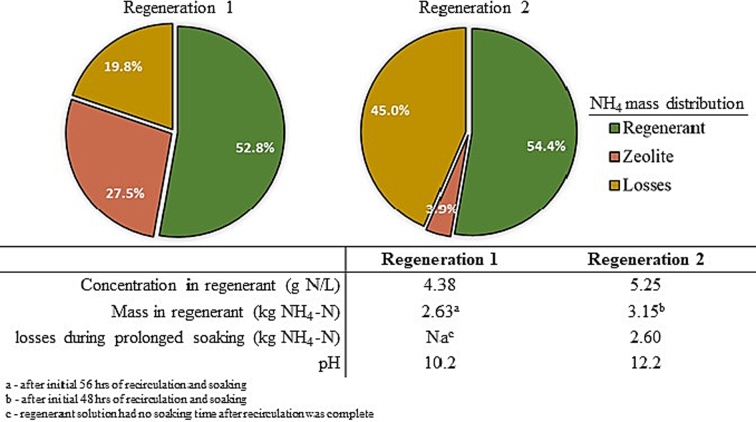
Ammonium mass balance during the two regeneration events conducted during the field trial.

The mass of NH_4_^+^ recovered in the regenerant was 2.63 kg NH_4_-N (4.38 g NH_4_-N/L) and 3.15 kg NH_4_-N (5.25 g NH_4_-N/L), with Regeneration 2 showing a slightly higher mass recovery than the first (Fig. 7). From a mass balance perspective, 52.8% and 54.4% of the estimated NH_4_-N sorbed onto the zeolite after Cycles 1 and 2 was recovered in the regenerant after Regeneration 1 and 2, respectively. The sorption capacity of cycle 2 was higher than cycle 1, thus yielding a similar percent recovery of ammonium in the regenerant but a higher mass recovery. The unaccounted portion of NH_4_-N may include ions lost as NH_3_ during recirculation, although the gas released was not quantified. Regeneration 2 had a long soaking time with regenerate solution, during which it was estimated that 2.60 kg or 45.0% of NH_4_-N was lost due to NH_3_ stripping out in a high pH solution. Even after 38 days of soaking, the pH of the solution remained high, at 10.2. Additionally, the turbidity in the regenerant after regeneration was completed was 4.30 NTU, decreasing to 0.51 NTU after the 38 days of static soaking, and increased to 134 NTU after the final 10th rinse with tap water.

## 4 Discussion

### 4.1 Zeolite regeneration procedure

Two successful onsite regeneration events were performed during the 1.5 year long field trial, with the second regeneration event showing an improvement in desorbing NH_4_-N from the zeolite clinoptilolite using a modified protocol. The second protocol used a higher concentration of NaCl, increased from 36 to 40 kg, the recirculation time of the regenerate increased from 15 to 30 h, and the soaking time during and after recirculation increased from 41 h to over 38 days. The additional contact time during the second regeneration improved the desorption of NH_4_-N from zeolite, however, it is unlikely that a full 38 days is necessary. Coupling the regeneration process with scheduled shutdown periods may be beneficial but not always possible, particularly when NSSS are used in settings where water treatment and water production are a necessity and any down time may be limited to only a few days. Unfortunately, zeolite granule samples were not taken immediately after recirculation ended during Regeneration 2, which would have revealed additional insight on desorption due to recirculation alone. The conductivity probe was also malfunctioning right before Regeneration 2 took place, further limiting the evaluation of the modifications to the regeneration procedure. However, the contact time with the regeneration did influenced the zeolite regeneration effectiveness. While increasing the NaCl may have helped drive the desorption kinetics during Regeneration 2, the data suggests that a much lower NaCl concentration can be used and still achieve NH_4_^+^ desorption into the bulk liquid, evident by the excess in residual NaCl in the final sample of Regeneration 1. As a conservative approach, the amount of Na^+^ utilized was 1.7 times higher than the minimum required to exchange all the original NH_4_-N sorbed onto the zeolite. Spikes in the conductivity and pH of the NCS effluent after Regeneration 1 signaled that poor mixing and insufficient rinsing led to these elevated values. This observation was addressed in the second regeneration by improving the physical mixing of the salt added to EQ3 and by increasing the bed volume rinses after regeneration to 10.

Since the conductivity of the rinse water during Regeneration 2 was not measured due to probe issues, a third regeneration was conducted in November 2020 after prolonged dormancy (data not shown) to quantify the rinsing needs. During this experiment, the regenerant included 20 kg of NaCl, reduced from 40 kg since the zeolite beds were anticipated to be near 50% exhausted. The results from this test showed that after the first rinse, 20 ± 0.81% of the conductivity was reduced (Fig. S2). By the 4th rinse, the conductivity reduction reached 99 ± 0.24%, suggesting that for a completely exhausted NCS using full strength regenerant, 6–7 bed volume rinses may be necessary. This was further confirmed by evaluating the bed volumes that passed through the NCS after the first regeneration to drop the conductivity from 4.6 mS/cm to 3.0 mS/cm, the stabilized conductivity of the BW. According to ISO 30500, conductivity is not an effluent water quality parameter for NSSS, but pH must remain between 6 and 9. The pH levels were also affected by rinsing procedure, however, even with only 4 rinses after Regeneration 1, the pH in the NCS effluent only rose above 9 for one sample point (pH = 9.07), suggesting that bed volume rinses can be further reduced. The rinsing procedure uses a significant amount of water and further work is required to optimize the procedure to either reduce the amount of rinsing needed or to capture and reuse a portion of the rinse water for future regenerations.

### 4.2 Ammonium recovery

In this study, BW consisting of toilet water from the CAB was used as the primary wastewater source. While there were various nitrogenous species that constituted the nitrogen portion of BW, the predominant form was as NH_4_-N. It was evident that the NH_4_-N loading increased when urinal YW was aggregated to the BW. The NH_4_-N loading rate onto the NCS varied depending on the nitrogen concentrations of the influent BW, but the observed concentration values were relatively similar to other studies (Knerr et al., 2011; Zhou et al., 2020). The addition of YW during the middle part of Cycle 1 until the end of Cycle 2, coupled with the increase in daily flow rates in the second half of Cycle 2, had a significant impact on the nitrogen load and therefore the rate of saturation of the zeolite beds.

Over 50% of the sorbed NH_4_-N was captured in the bulk liquid after each regeneration, equivalent to nearly 5 g/L of NH4-N recovered for reuse. The high pH of the regenerant allowed for a portion of the sorbed NH_4_-N to escape as NH_3(g)_ during recirculation and even during prolonged soaking during Regeneration 2. This high pH recycle-batch approach, instead of a continuous flow system, was undertaken to decrease any resorbing of NH_4_-N onto the zeolite beds during the recirculation process and to reduce regenerant waste production. Volatilizing a partial amount of NH_4_-N may be a consequence for small-scale systems to reduce the amount of brine generated onsite, however, the regeneration procedure in this study was able to recover at minimum 53% of the sorbed NH_4_-N after the recirculation period. One caveat is that the saturation of zeolite did not consider other competing cationic compounds adsorbing to the surface simultaneously. It is well documented that other relevant cations such as K^+^, Na^+^, Ca^2+^, and Mg^2+^ present in wastewater can compete with NH_4_^+^ for surface sites on the zeolite (Ames, 1960). These particular ions may be present in BW at much higher concentrations than typical domestic wastewater, decreasing the available surface sites for NH_4_^+^ uptake, and underestimating the NH_4_-N recoverability. Additionally, it is also possible that biological activity may have contributed to a partial loss in NH_4_-N within the NCS during each cycle, consequently, causing a minor overestimation of the amount of recoverable NH_4_-N on the zeolite.

For our study, an estimated 600 L of regenerant waste was produced during each regeneration, equivalent to 1.2 bed volumes. By comparison, laboratory-based studies have used anywhere between 5 and 20 bed volumes of regenerant (Guo et al., 2007). However, the generated brine from this study still contained excess Cl^−^ and residual Na^+^ ions along with the desorbed NH_4_^+^ ions. There are various new techniques currently being studied to separate and recover the NH_4_-N from the brine, particularly desalination approaches that include capacitive deionization, membrane distillation, forward osmosis, and battery deionization (Huo et al., 2020; Ray et al., 2020; Son et al., 2020; Wimalasiri et al., 2015). The limitation in using any of these approaches will be the efficiency in separating NH_4_^+^ from the brine in the presence of competing monovalent cations, in particular, residual Na^+^ ions from the regenerant.

### 4.3 Regeneration frequency

The first regeneration event did not occur until about 1 year from when NG first began operations. However, the second regeneration was needed only four months after the first regeneration. This was highly influenced by the composition of the incoming wastewater and the added nitrogen source in YW as well as the lower desorption efficiency from Regeneration 1. One method to extend the time between zeolite regenerate events, beyond optimizing the desorption efficiency, is to employ urine diversion upstream. While this may not be feasible at all locations, maintaining a steady nitrogen loading rate allows the operators to better predict when the zeolite beds may be near saturation. In this study, when urine diversion was employed and the NG was operated at consistent flow rates, the loading rate of NH_4_-N remained low and consistent, near 0.1 kg m^-3^ d^-1^. As a conservative approach, the zeolite beds in the NCS would require semi-annual regenerations. Regeneration cycles have been estimated as high as 10–20 for zeolite without much loss to regeneration efficiency, which would entail that the zeolite beds of the NCS would need full replacement after 5–10 years (Katsou et al., 2011; Neag et al., 2019; Zhang et al., 2017). Guo et al. (2007) observed consistently high NH_4_^+^ removal (>99%) and recovered 4 g N/L even after 5 regeneration cycles. The major concern with reusing zeolite for extended regeneration cycles is the loss of zeolite mass by disintegration, although, Zhang et al. (2017) documented that the surface morphology and chemical composition of zeolite clinoptilolite was largely unchanged even after 20 regeneration cycles when using NaClO-NaCl as a regenerant solution. A caveat to these results is that they have largely been conducted with synthetic media under controlled environments. Natural zeolites inherently contain impurities which may be critical to their structural integrity. High temperatures and acidic conditions can remove these impurities (Hartman and Fogler, 2007). During Regeneration 2 of this study, the turbidity of the regenerant increased significantly from 4.30 NTU at the end of the initial 48 h recirculation period to 134 NTU after the 38 days of prolonged static soaking and 10 bed volume rinses. The results signal that the prolonged exposure to an alkaline solution and subjection to South Africa's summer temperatures in an enclosed tricon can lead to potential disintegration of the granules. The long-term effects of extreme environmental conditions and wastewater composition will be integral in understanding the risk of weakening the structure's framework over time. Further studies at the pilot-scale will be needed to quantify the long-term effects of treating BW on regeneration frequency and efficiency. Nonetheless, the cost of natural zeolite is very low by which it could be replaced more frequently if needed.

### 4.4 System monitoring

The NH_4_-N removal performance in the NCS began to drop when the zeolite's NH_4_-N sorption reached 55–60% of the estimated maximum capacity. During this period, NH_4_-N breakthrough in the post-NCS sample was observed. This suggests that at nearly half of the capacity of NH_4_-N adsorption, the zeolite is nearing saturation and signals that a regeneration of the beds should take place soon. ISO 30500 outlines that TN removal must be greater than 70% which limits the extent to which the zeolite can be exhausted to before treatment goals are no longer met. The NH_4_-N sorption rate by the NCS ranged from 0.05–0.11 g/kg-d during normal operations with BW only, suggesting that when the NCS reaches 50% capacity, the treatment performance of the NCS will drop below 70% removal within 50–100 days. Reaching this capacity will be dependent on the daily flow rates and the wastewater composition entering the system. Real-time monitoring of NH_4_-N would allow operators to better predict when regeneration is needed and when the effluent quality could be compromised. An inline or portable NH_4_^+^ ion selective probe can be a useful tool to determine when regeneration is needed. From a monitoring perspective, when NSSS are deployed to sites, it will be imperative to keep sample testing and outside laboratory analysis to a minimum. Resources need to be directed towards measuring regulated parameters (COD, TSS, pH, TN, TP, and pathogens), and any additional testing would require added costs for communities that are already strained financially. As this study illustrates, a zeolite ion exchange technology can be successfully implemented in NSSS while requiring minimal monitoring to meet regulatory criteria.

### 4.5 Field test limitations

Field testing the NG system was vital in understanding the zeolite performance when treating high-strength BW and developing a protocol for onsite regeneration under actual environmental constraints. Several of these constraints include field operational parameters such as the variability in influent wastewater composition and the daily flow rates of NG, with flow rates being heavily dependent upon the operator's availability onsite and down time due to troubleshooting and maintenance of the system. As is the nature of field trials, there were unanticipated issues, such as pump malfunction during regeneration events limiting the recirculation times, probe malfunction, constraints with sample acquisitions, and inadequate dissolution of the NaCl prior to regeneration. Other constraints include environmental parameters such as temperature, pH, conductivity, and ionic strength, each having a different influence on the sorption rate and capacity depending on the morphology and size of the zeolite used, and whether it is natural or synthetic. Nonetheless, the study was able to generate invaluable data about procedural steps for onsite regeneration and modifications that can be undertaken to shorten the regeneration time, minimize the generation of waste, and recover NH_4_-N.

## 5 Conclusions

For small-scale decentralized treatment systems, the use of ion exchange media like zeolite is a feasible method for reliably removing NH_4_-N from high-strength wastewater like undiluted BW treated by NSSS. On a practical level, this ion exchange process can handle variable nitrogen loads due to daily flow fluctuations caused by intermittent use and periods of dormancy which allows for minimal oversight of the NCS during regular operations. While NH_4_-N can be removed and recovered at high efficiencies, the regeneration process itself can be time consuming, currently taking up to 5 days from start to end and generates an undesired brine solution, albeit, at much smaller quantities than most ion exchange regeneration processes. The regeneration process time can be cut down further with modifications to the hydraulic design to improve contact time. However, NH_4_^+^ ions will still require separation from the brine solution and must be further processed. Future work should focus on the separation of NH_4_^+^ from the brine solution and potentially recovering Na^+^ for reuse as a regenerate ion as a means for sustainably managing nitrogen and other recoverable ions.

## CRediT authorship contribution statement

**C.J. Castro:** Conceptualization, Project administration, Data curation, Formal analysis, Writing – original draft, Writing – review & editing. **H.Y. Shyu:** Investigation, Methodology, Writing – review & editing. **L. Xaba:** Investigation, Resources, Writing – review & editing. **R. Bair:** Conceptualization, Project administration, Writing – review & editing. **D.H. Yeh:** Conceptualization, Project administration, Funding acquisition, Supervision, Writing – review & editing.

## Declaration of competing interest

The authors declare the following financial interests/personal relationships which may be considered as potential competing interests: Daniel Yeh and Robert Bair are named inventors on patent applications for technologies related to the NEWgenerator. USF is the assignee on the patents, and has licensed technologies related to the NEWgenerator to companies in India and South Africa.

Daniel Yeh and Robert Bair are co-founders of BioReNEW, Inc.

## Appendix A Supplementary data

Supplementary material Image 1
