# Supplementary material for: Performance and onsite regeneration of natural zeolite for ammonium removal in a field-scale non-sewered sanitation system
Source: Sci Total Environ. 2021 Jul 1;776:145938. doi: 10.1016/j.scitotenv.2021.145938 (PMC8111385; doi:10.1016/j.scitotenv.2021.145938)
Supplement: Supplementary file 1 — Supplementary material [file mmc1.docx]

**Performance of natural zeolite for onsite ammonium recovery in a field-scale non-sewered sanitation system**

Supplemental Information

Table S1. Summary of nitrogenous species concentrations across the treatment train during each loading cycle.

|  | **TN (mg N/L)** | | | | **NH_4_-N (mg N/L)** | | | | **NO_3_-N (mg N/L)** | | | |
| --- | --- | --- | --- | --- | --- | --- | --- | --- | --- | --- | --- | --- |
|  | *mean ± std dev.* | *min* | *max* | *n* | *mean ± std dev.* | *min* | *max* | *n* | *mean ± std dev.* | *min* | *max* | *n* |
| **Cycle 1** | | | | | | | | | | | | |
| *NG Influent* | 250 ± 92 | 88 | 440 | 33 | 180 ± 94 | 11 | 360 | 34 | 2.1 ± 1.2 | 0.0 | 5.7 | 30 |
| *Reactor* | 300 ± 120 | 100 | 690 | 33 | 190 ± 87 | 10 | 360 | 34 | 2.7 ± 1.9 | 0.0 | 7.6 | 30 |
| *Permeate* | 230 ± 91 | 63 | 380 | 32 | 200 ± 98 | 10 | 360 | 34 | 1.4 ± 1.1 | 0.0 | 4.5 | 30 |
| *NCS* | 60 ± 90 | 0 | 280 | 32 | 55 ± 85 | 0 | 270 | 34 | 0.60 ± 0.58 | 0.0 | 1.8 | 30 |
| *Chlorination* | 57 ± 87 | 0 | 280 | 32 | 54 ± 83 | 0 | 270 | 34 | 0.59 ± 0.65 | 0.0 | 2.2 | 30 |
| **Cycle 2** | | | | | | | | | | | | |
| *NG Influent* | 350 ± 72 | 270 | 540 | 15 | 300 ± 71 | 200 | 480 | 15 | 2.5 ± 1.0 | 1.5 | 5.5 | 15 |
| *Reactor* | 490 ± 110 | 360 | 730 | 15 | 300 ± 71 | 200 | 480 | 15 | 4.8 ± 2.1 | 2.1 | 8.8 | 15 |
| *Permeate* | 320 ± 110 | 32 | 490 | 15 | 320 ± 83 | 140 | 470 | 15 | 1.9 ± 0.48 | 0.8 | 2.6 | 15 |
| *NCS* | 110 ± 89 | 33 | 280 | 15 | 110 ± 84 | 31 | 260 | 15 | 1.6 ± 0.79 | 0.0 | 3.4 | 14 |
| *Chlorination* | 100 ± 96 | 17 | 280 | 15 | 105 ± 93 | 8.9 | 270 | 15 | 1.7 ± 0.74 | 0.0 | 2.5 | 14 |
| **Cycle 3** | | | | | | | | | | | | |
| *NG Influent* | 280 ± 66 | 240 | 390 | 6 | 250 ± 60 | 210 | 360 | 6 | 1.7 ± 0.89 | 0.0 | 2.5 | 6 |
| *Reactor* | 600 ± 122 | 480 | 790 | 6 | 250 ± 60 | 210 | 360 | 6 | 7.6 ± 2.7 | 3.5 | 11 | 6 |
| *Permeate* | 370 ± 120 | 250 | 530 | 6 | 350 ± 110 | 250 | 520 | 6 | 1.6 ± 0.85 | 0.0 | 2.4 | 6 |
| *NCS* | 36 ± 62 | 0 | 160 | 6 | 31 ± 57 | 0 | 150 | 6 | 1.3 ± 0.94 | 0.0 | 2.8 | 6 |
| *Chlorination* | 45 ± 79 | 1.6 | 200 | 6 | 38 ± 71 | 0 | 180 | 6 | 1.6 ± 1.0 | 0.0 | 3.2 | 6 |


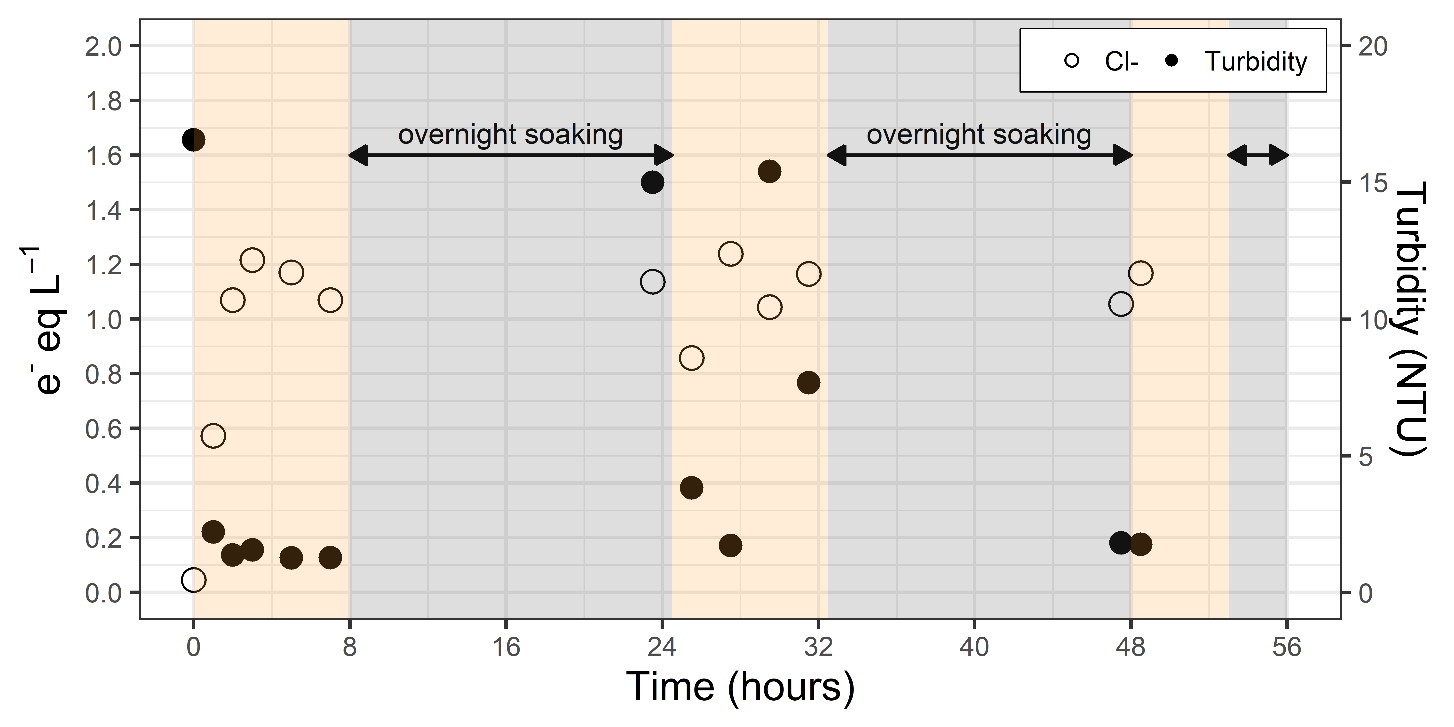


Figure S1. Electron equivalents of Cl^-^ ions and turbidity as additional water quality measurements of the regenerant during regeneration 1. Note: grey areas represent periods of static and/or overnight soaking and orange areas are periods of regenerant recirculation.


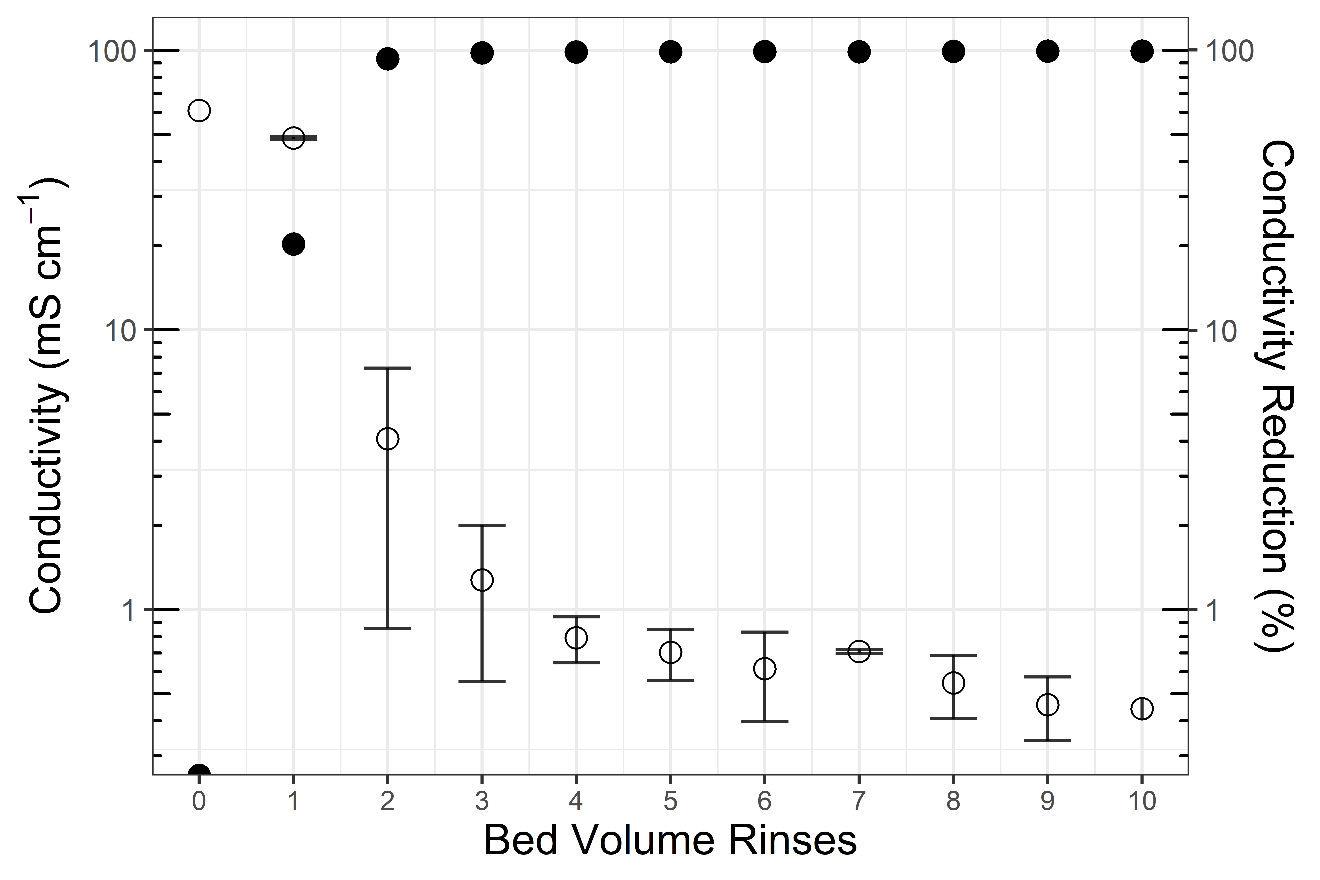


Figure S2. The average conductivity (open circle) and average percent reduction (closed circle) during consecutive rinses of the zeolite beds with tap water during a third regeneration executed in November of 2020 after a shutdown period due to COVID-19 pandemic. Error bars represent the standard deviation between both zeolite beds.
